# Risks of occupational illnesses among health workers providing care to patients with COVID-19: an integrative review*

**DOI:** 10.1590/1518-8345.4895.3455

**Published:** 2021-06-28

**Authors:** Edwing Alberto Urrea Vega, Liliana Antoniolli, Andréia Barcellos Teixeira Macedo, Jéssica Morgana Gediel Pinheiro, Thayane Martins Dornelles, Sônia Beatriz Cócaro de Souza

**Affiliations:** 1 Universidade Federal do Rio Grande do Sul, Porto Alegre, RS, Brazil.; 3 Hospital Moinhos de Vento, Programa de Apoio ao Desenvolvimento Institucional do Sistema Único de Saúde (PROADI-SUS), Porto Alegre, RS, Brazil.; 4 Universidade Federal do Rio Grande do Sul, Departamento de Enfermagem Médico-Cirúrgica, Porto Alegre, RS, Brazil.

**Keywords:** Coronavirus Infections, Occupational Exposure, Health Personnel, Allied Health Personnel, Nurses, Physicians, Infecções por Coronavirus, Exposição Ocupacional, Pessoal de Saúde, Pessoal Técnico de Saúde, Enfermeiras e Enfermeiros, Médicos, Infecciones por Coronavirus, Exposición Profesional, Personal de Salud, Técnicos Medios en Salud, Enfermeras y Enfermeros, Médicos

## Abstract

**Objective:**

to analyze evidence concerning the risks of occupational illnesses to which health workers providing care to patients infected with COVID-19 are exposed.

**Method:**

integrative literature review conducted in the following online databases: Medical Literature Analysis and Retrieval System Online (MEDLINE/PubMed), Web of Science (WoS), Excerpta Medica Data-Base (EMBASE), Cumulative Index to Nursing and Allied Health Literature (CINAHL) and Scopus (Elsevier). Original articles published between November 2019 and June 2020, regardless of the language written, were included. A descriptive analysis according to two categories is presented.

**Results:**

the sample is composed of 19 scientific papers. Most were cross-sectional studies with an evidence level 2C (n=17, 90%) written in English (n=16, 84%). The primary thematic axes were risk of contamination and risk of psycho-emotional illness arising from the delivery of care to patients infected with COVID-19.

**Conclusion:**

the review presents the potential effects of providing care to patients with COVID-19 on the health of workers. It also reveals the importance of interventions focused on the most prevalent occupational risks during the pandemic. The studies’ level of evidence suggests a need for studies with more robust designs.

## Abstract (pt)

**Objetivo:**

analisar as evidências sobre os riscos de adoecimento ocupacional aos quais estão expostos os profissionais de saúde que cuidam de pacientes acometidos pela COVID-19.

**Método:**

revisão integrativa da literatura realizada por meio de busca on-line nas bases de dados *Medical Literature Analysis and Retrieval System Online* (MEDLINE/PubMed), *Web of Science* (WoS), *Excerpta Medica Data-base* (EMBASE), *Cumulative Index to Nursing and Allied Health Literature* (CINAHL) e *Scopus* (Elsevier). Incluíram-se artigos originais, publicados entre novembro de 2019 e junho de 2020, sem restrições de idioma. A análise descritiva dos resultados é apresentada em duas categorias.

**Resultados:**

a amostra constituiu-se de 19 produções científicas com predomínio da língua inglesa (n=16, 84%) e estudos de corte transversal, com nível de evidência 2C (n=17, 90%). Os estudos mostraram, como principais eixos temáticos, o risco de contaminação e o risco de adoecimento psicoemocional no atendimento a pacientes acometidos pela COVID-19.

**Conclusão:**

a revisão mostrou os potenciais efeitos sobre a saúde dos profissionais durante o atendimento de pacientes acometidos pela COVID-19. Evidenciou-se a importância da implementação de estratégias de intervenção focadas nos riscos ocupacionais mais prevalentes durante a pandemia. O nível de evidência dos estudos sugere a necessidade de desenvolvimento de pesquisas com delineamentos mais robustos.

## Abstract (es)

**Objetivo:**

analizar las evidencias sobre los riesgos de enfermedad ocupacional a los cuales están expuestos los profesionales de la salud que cuidan de pacientes afectados por la COVID-19.

**Método:**

revisión integradora de la literatura realizada a través de búsqueda online en las bases de datos *Medical Literature Analysis and Retrieval System Online* (MEDLINE/PubMed), *Web of Science* (WoS), *Excerpta Medica Data-base* (EMBASE), *Cumulative Index to Nursing and Allied Health Literature* (CINAHL) y *Scopus* (Elsevier). Fueron incluidos artículos originales, publicados entre noviembre de 2019 y junio de 2020, sin restricciones de idioma. El análisis descriptivo de los resultados se presenta en dos categorías.

**Resultados:**

la muestra fue constituida por 19 producciones científicas con predominio del idioma inglés (n=16, 84%) y estudios de corte transversal con nivel de evidencia 2C (n=17, 90%). Los estudios mostraron como principales ejes temáticos el riesgo de contaminación y riesgo de enfermedad psicoemocional, en la atención a pacientes afectados por COVID-19.

**Conclusión:**

la revisión mostró los potenciales efectos sobre la salud de los profesionales durante la atención de pacientes afectados por COVID-19. Se evidenció la importancia de implementar estrategias de intervención, enfocadas en los riesgos ocupacionales más prevalentes durante la pandemia. El nivel de evidencia de los estudios sugiere la necesidad de desarrollar investigaciones con delineamientos más robustos.

## Introduction

In 2020, a pandemic caused by the novel coronavirus (COVID-19) devastated the world, causing thousands of deaths. COVID-19 originated in China at the end of 2019 and rapidly spread worldwide. The disease may cause a pandemic respiratory syndrome called severe acute respiratory syndrome coronavirus 2 (SARS-CoV-2), requiring, in some cases, critical or intensive care arising from more complex and severe conditions^(^ 1 ^)^.

The first cases in Brazil were recorded in February 2020, and the number of infected people and deaths due to COVID-19 has only increased. Brazil currently ranks second with the highest number of cases, lethality of 2.9% and mortality rate of 76.7%, only behind the United States; 161,106 deaths have been counted so far^(^ 2 ^)^.

Many countries reported a collapse of their health systems at a certain point of the pandemic and experienced a lack of personnel and material and physical resources to care for all the individuals infected with COVID-19^(^ 3 ^)^. Additionally, the virus’ high transmissibility and spreading rate, higher than that estimated at the beginning of the pandemic, increased the risk of occupational exposure and illness, especially among health workers assisting infected patients^(^ 4 ^)^.

Becoming immune after being infected with COVID-19 is not certain, and vaccines are still undergoing clinical tests. While immunological barriers are not consolidated and ensured by science, health workers, especially those in direct contact with patients infected with COVID-19, can only resource to physical barriers such as personal protective equipment (PPEs), social distancing, and hand and environment sanitation to protect from and minimize the risk of contagion^(^ 5 ^-^ 6 ^)^.

The different working conditions of the various productive sectors, the health history of employees, conflict of roles, problems faced in interpersonal relationships, and biological and/or psychosocial risks arising from the professional activity are maximized by long working hours, lack of PPEs, and potential physical, emotional or mental distress imposed to health workers during the pandemic. As reported by a study conducted in Spain^(^ 4 ^)^, health managers should consider these factors a sign to intensify strategies intended to promote occupational health and prevent diseases.

It is crucial to preserve workers’ health to minimize the dissemination of COVID-19 and manage the effects of contamination, which reflect on hospital facilities and primary health care services^(^ 7 ^-^ 8 ^)^. Therefore, occupational health strategies are essential to provide protective barriers and provide support and integral care, including psycho-emotional care. The establishment of effective actions requires identifying the occupational risks to which health workers are exposed^(^ 9 ^)^.

Thus, this investigation is based on the importance of producing and aggregating knowledge by searching the scientific literature to ground health promotion activities, minimizing the risk of occupational illnesses among health workers exposed to patients with confirmed or suspected COVID-19 infection. This study’s objective was to analyze evidence concerning occupational risks to which health workers providing care to patients with COVID-19 are exposed.

## Method

This literature Integrative Review (IR) was conducted through six distinct stages: 1) establishment of the guiding question; 2) search and selection of primary studies; 3) extraction data from primary studies; 4) critical assessment of the primary studies; 5) synthesis of results; and 6) presentation of results^(^ 10 ^)^.

The guiding question was established according to the PICO strategy (P-population, I-Intervention, C-comparison, and O-outcome)^(^ 11 ^)^ to increase the probability of finding evidence in secondary sources that meet the assumption of Evidence-Based Practice. Thus, the following guiding question was established: “What are the occupational risks to which health workers providing care to patients infected with COVID-19 are exposed?”

The search strategy used to meet the integrative review’s objectives includes controlled terms combined with boolean operators adapted to the specificities of each database. See details in Figure 1.

**Figure 1:** Descriptors used in the IR according to the PICO* strategy and boolean operators. Porto Alegre, RS, Brazil, 2020

PICO*	Search terms	Controlled descriptors
P- Population	(Health Care Provider) OR (Health Care Providers) OR (Healthcare Provider) OR (Healthcare Providers) OR (Healthcare Worker) OR (Healthcare Workers) OR (Personnel, Health) OR (Provider, Health Care) OR (Provider, Healthcare) OR (Providers, Health Care) OR (Providers, Healthcare) OR (Health AND Worker) OR (Personnel AND Health) OR (Health Care AND Provider) OR (Healthcare AND Worker) (Allied Health Professional) OR (Allied Health Professionals) OR (Assistant, Healthcare) OR (Assistants, Healthcare) OR (Health Personnel, Allied) OR (Health Professional, Allied) OR (Health Professionals, Allied) OR (Healthcare Assistant) OR (Healthcare Assistants) OR (Healthcare Support Worker) OR (Healthcare Support Workers) OR (Paramedic) OR (Paramedical Personnel) OR (Paramedics) OR (Personnel, Allied Health) OR (Personnel, Paramedical) OR (Population Program Specialist) OR (Population Program Specialists) OR (Professional, Allied Health) OR (Professionals, Allied Health) OR (Program Specialist, Population) OR (Program Specialists, Population) OR (Specialist, Population Program) OR (Specialists, Population Program) OR (Support Worker, Healthcare) OR (Support Workers, Healthcare) OR (Worker, Healthcare Support) OR (Workers, Healthcare Support) (Nurses) OR (Nurse) OR (Nurse, Registered) OR (Nurses, Registered) OR (Nursing Personnel) OR (Personnel, Nursing) OR (Registered Nurse) OR (Registered Nurses) OR (nurs) Physicians	Healthcare workers Healthcare Support Workers Nurses Physicians
I- Intervention/area of interest	(COVID-19) OR (2019 novel coronavirus Pneumonia) OR (2019-novel coronavirus Pneumonia) OR (2019 novel coronavirus Epidemic) OR (2019 novel coronavirus Outbreak) OR (2019 novel coronavirus Pandemic) OR (2019-nCoV Acute Respiratory Disease) OR (2019-nCoV Epidemic) OR (2019-nCoV Outbreak) OR (2019-nCoV Pandemic) OR (2019-nCoV Pneumonia) OR (2019-novel coronavirus (2019-nCoV) Infection) OR (2019­new coronavirus Epidemic) OR (2019­20 China Pneumonia Outbreak) OR (2019­20 Wuhan coronavirus Outbreak) OR (COVID-19) OR (Coronavirus Infection) OR (Infection, Coronavirus) OR (Infections, Coronavirus) OR (MERS (Middle East Respiratory Syndrome)) OR (Middle East Respiratory Syndrome) OR (Novel Coronavirus Pneumonia) OR (Wuhan Seafood Market Pneumonia) OR (Wuhan coronavirus Epidemic) OR (Wuhan coronavirus Infection) OR (Wuhan coronavirus Outbreak) OR (Wuhan coronavirus Pandemic) OR (Wuhan coronavirus Pneumonia)	Coronavirus infections
C- Comparison	Does not apply	Does not apply
O- Outcome	(Exposure, Occupational) OR (Exposures, Occupational) OR (Occupational Exposures)	Occupational exposure

* PICO = P- population; I- Intervention/area of interest; C- comparison; O- outcome

The search was conducted between March and June 2020 on the following databases: Medical Literature Analysis and Retrieval System Online (MEDLINE via PubMed); Web of Science (WoS); Excerpta Medica Data-Base (EMBASE); Cumulative Index to Nursing and Allied Health Literature (CINAHL), and Scopus (Elsevier). The studies were accessed using the periodicals portal of the Coordination for the Improvement of Higher Education Personnel (CAPES).

Inclusion criteria were: primary articles addressing the occupational exposure of health workers providing care to patients with confirmed or suspected COVID-19 infection, with publication starting November 2019 and associated with the first outbreak of the disease in Wuhan, in the Hubei province, China, regardless of the language. Exclusion criteria were: theses, dissertations, editorials, reviews, manuals, protocols, book chapters, reflections, opinion reports, or experts’ comments. Duplicated versions were deleted.

The initial search in the electronic databases disregarded duplicated papers and included an analysis of titles and abstracts to ensure the papers addressed the guiding question. A form was developed in the Microsoft Excel 2013® to extract data from the primary studies: author(s), title, abstract, study’s objective, year, country of publication, design, main results, conclusions, limitations, and level of evidence.

The Oxford Centre for Evidence-based Medicine^(^ 12 ^)^ classification was used to analyze the level of evidence: 1A – systematic review (with homogeneity) of randomized controlled clinical trials; 1B - randomized controlled clinical trials with a narrow confidence interval; 1C – “all or none” therapeutic results; 2A – systematic reviews of cohort studies; 2B – cohort studies (including low-quality randomized clinical trials); 2C – observation of therapeutic outcomes or ecological studies; 3A – systematic review (with homogeneity) of case-control studies; 3B – case-control studies; 4 –case reports (including cohort or low-quality case-control); 5 – expert opinion without explicit critical appraisal or based on physiology, bench research or “first principles”.

Two researchers independently selected, extracted data, and critically assessed the full texts of the primary studies selected. A calibration process was performed between the researchers before critical analysis and synthesis of the results to seek consensus on relevant concepts for the review’s central theme. Consensus was reached with the support of a third evaluator in case of disagreement.

The studies’ critical analysis and synthesis are descriptively presented using a synoptic table to facilitate the identification and objectively compare conflicting or different findings and summarize similar results answering the guiding question. When ordering and classifying the sample according to semantic and theoretical similarity, other factors imbricated to the risk of occupational illness were considered, from which two thematic categories emerged: risk of contamination and occupational exposure of health workers providing care to patients infected with COVID-19 and risk of psycho-emotional illness of health workers providing care to patients infected with COVID-19.

The authorship of the sources used was respected according to Law 9,610 from February 19th 1998 that regulates copyrights in Brazil^(^ 13 ^)^.

## Results

A total of 1,656 scientific papers were identified, and 1,617 were eliminated after applying inclusion and exclusion criteria. A total of 97 were duplicated, while the titles and abstracts of 1,520 papers were not pertinent due to the following reasons: were not an article, not research, did not address the theme, or did not answer the guiding question.

**Figure 2:**
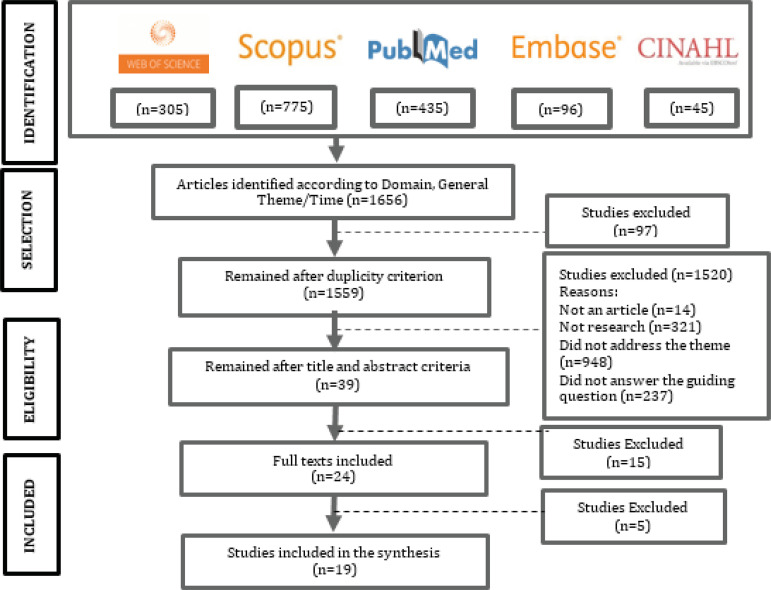
Flowchart of primary studies based on PRISMA*. Porto Alegre, RS, Brasil, 2020 *PRISMA = Preferred Reporting Items for Systematic Review and Meta-Analyses

After this stage, the full texts of 39 papers remained. The researchers excluded 15 of these because they did not answer the guiding question, while a consensus was obtained to exclude another five papers. Finally, 19 papers remained, as shown in the flowchart presented in Figure 2.

Regarding the characterization of the studies included in the final sample, the papers were all published in 2020; most studies addressing the theme were published in China (68%), followed by Germany (5%), Singapore (5%), Paraguay (5%), Turkey (5%), and Israel (5%), while one was a multicenter study (5%). Most studies were written in English (84%), followed by Chinese (11%), and Spanish (5%). Concerning the studies’ designs and level of evidence, according to the Oxford classification, we have: cross-sectional studies – evidence level 2C (90%), one cohort study – level of evidence 2B (5%), and a case series – evidence level 4 (5%).

Figure 3 presents a synthesis of the main elements extracted from the studies composing the final sample, distributed according to the thematic categories.

**Figure 3:** Characterization of the primary studies included in the integrative review according to the primary author, periodical, database, year of publication, design, sample, country of origin, objective, main results, and level of evidence. Porto Alegre, RS, Brazil, 2020

Category 1. Risk of contamination and occupational exposure among health workers providing care to patients infected with COVID-19^¶^.				
Primary author / Journal / Database / Year	Design / Sample / Country	Objective	Result	LE^¶¶¶^
Ran L, et al./ *Clinical Infectious Diseases* /EMBASE^*^/(2020)^(^ 14 ^)^	Retrospective cohort study /72 physicians and nurses / China	To determine the risk factors and behaviors associated with COVID-19^¶^ among health workers.	The group of high-risk workers was 2.13 times more likely of developing COVID-19^¶^ than the group of workers in general (RR^§§^ raw=2.13, ^||||^95% CI: 1.45-3.95, ^¶¶^p<0.05).	2B****
Liu M, et al./ *Chinese Journal of Tuberculosis and Respiratory Diseases* /MEDLINE-Pubmed^†^/(2020)^(^ 15 ^)^	Retrospective case series - 30 participants (22 physicians and 8 nurses)/China	To investigate the clinical characteristics of the medical team with the novel coronavirus pneumonia (NCP)^**^.	The average time of contact was 12 hours (7.16), and the average accumulated time of contact was 2 hours (1.5,2.7). The primary manifestation of 83.33% of the participants was cough and dry cough, in most cases, approximately one week after admission. Later, 14 (46.67%) participants experienced difficulty breathing.	4^††††^
Korth J, et al./ *Journal of Clinical Virology* / SCOPUS^‡^/ (2020)^(^ 16 ^)^	Prospective cross-sectional study/ 316 health workers /Germany	To determine the seroprevalence of SARS-CoV-2^††^ among health workers from the Essen University Hospital, Germany.	SARS-CoV-2^††^-*** IgG antibodies were detected in 5 of the 316 (1.6%) individuals.	2C^‡‡‡‡^
Delgado D, et al./ *International Journal of Environmental Research and Public Health* / SCOPUS^‡^/ (2020)^(^ 17 ^)^	Cross-sectional study/ 936 health workers/ Latin American (Spanish speaking countries).	To assess the context and personal safety perceptions of health workers from Latin America countries during the COVID-19^¶^ outbreak.	Most participants (699; 74.7%) accessed the COVID-19^¶^ diagnosis and treatment algorithms, while 237 (25,3%) did not.	2C^‡‡‡‡^
Ong JJY, et al./ *Headache: The Journal of Head and Face Pain* /SCOPUS^‡^/(2020)^(^ 18 ^)^	Cross-sectional study/ 158 health workers/ Singapore	To determine risk factors associated with headaches associated with new PPE^‡‡^ and perceived impact of these headaches on personal health and work performance.	The participants with a diagnosis of preexisting primary headache (OR^†††^ = 3.44; ^||||^95% CI 1.14-10.32; ^¶¶^p=0.013) and those working in the emergency department (OR^†††^=2.39, ^||||^95%CI 1.05-5.47; ^¶¶^p=0.019) were more likely to develop headaches associated with the prolonged use of N95 face masks and goggles.	2C^‡‡‡‡^
**Category 2**. Risk of psycho-emotional illness among health workers providing care to patients infected with COVID-19^¶^.				
Li Z, et al./ *Brain, Behavior, and Immunity* /EMBASE*/(2020)^(^ 19 ^)^	Descriptive cross-sectional study/ 740 individuals (214 public in general and 526 nurses)/China	To identify and provide an intervention for vicarious trauma in its initial stage.	The scores obtained by nurses working in the front line concerning vicarious trauma, including scores concerning physiological and psychological outcomes, were significantly lower than that obtained by nurses not working in the front line (^¶¶^p <0.001) and the public in general (^¶¶^p<0.001).	2C^‡‡‡‡^
Lai J, et al./ *JAMA Network Open* /CINAHL^§^/(2020)^(^ 20 ^)^	Cross-sectional cohort study /1,257 health workers (493 physicians and 764 nurses)/China	To assess the magnitude of mental health outcomes and associated factors among health workers providing care to patients infected with COVID-19^¶^ in China.	A considerable portion of participants presented symptoms of depression (634 [50.4%]), anxiety (560 [44.6%]), insomnia (427 [34.0%]), and anguish (899 [71.5%]).	2C^‡‡‡‡^
Kang L, et al./ *Brain, Behavior, and Immunity* /EMBASE*/(2020)^(^ 21 ^)^	Cross-sectional cohort study /994 health workers (183 physicians and 811 nurses)/China	To verify the mental health condition of the medical and nursing staff in Wuhan, the efficacy of psychological care, and psychological care needs.	36% of the workers presented mental disorders below the average threshold; 34.4% presented mild disorders; 22.4% presented moderate disorders; and 6.2% presented severe disorders. No significant differences were found regarding demographic data.	2C^‡‡‡‡^
Huang JZ, et al./ *Chinese Journal of Industrial Hygiene and Occupational Diseases* /EMBASE*/(2020)^(^ 22 ^)^	Cross-sectional cohort study/230 health workers (70 physicians and 160 nurses)/China	To investigate the medical staff's mental health working in the front line against the COVID-19^¶^pandemic and provide a theoretical ground for psychological intervention.	The incidence of anxiety among the nurses was higher than among the physicians [26.88% *vs.* 14.29% ^¶¶^p=0.039]. The incidence of stress in the health staff was 27.39%.	2C^‡‡‡‡^
Primary author / Journal / Database / Year	Design / Sample / Country	Objective	Result	LE^¶¶¶^
Xiao H, et al./Medical Science Monitor: International Medical Journal of Experimental and Clinical Research/ MEDLINE-Pubmed†/ (2020)^(^ 23 ^)^	Cross-sectional observational study/180 participants (nurses and physicians)/China	Structural equation modeling was used to determine the effect of social support on sleep quality and function among the health workers who provided care to patients with COVID-19^¶^ in January and February 2020 in Wuhan.	The results show that the social support provided to the medical team negatively affected (decreased) their anxiety and stress levels and positively affected their self-efficacy, though it did not directly improve sleep quality. The team's anxiety levels significantly affected stress levels and significantly decreased their self-efficacy and sleep quality.	2C^‡‡‡‡^
Xiao X, et al./Journal of Affective Disorders/SCOPUS‡/(2020)^(^ 24 ^)^	Multicenter cross-sectional study/958 participants/China	To assess stress levels and psychological morbidities such as anxiety and depression among health workers during the COVID-19^¶^outbreak.	The results showed that different positions (junior, intermediate and senior) (^¶¶^p=0.02) and professional experience in years (fewer than 5 years, 6-10 years, and more than 11 years) (^¶¶^p=0.048) affected the health workers' stress levels.	2C^‡‡‡‡^
Wu Y, et al./Journal of Pain and Symptom Management/EMBASE*/(2020)^(^ 25 ^)^	Cross-sectional study/220 participants/China	To compare the frequency of burnout between physicians and nurses working in the front line and those working in standard wards.	The frequency of burnout is significantly lower among front line workers than those working in standard wards (13% vs. 39%; ^¶¶^p<0.0001). The frequency of a low level of personal achievement is lower in the front line group than in the standard wards (39% vs. 61%; ^¶¶^p=0.002).	2C^‡‡‡‡^
Samaniego A, et al./Revista Interamericana de Psicología/SCOPUS‡/ (2020)^(^ 26 ^)^	Cross-sectional study /126 health professionals /Paraguay	To determine the prevalence of depression, anxiety, insomnia, anguish, and compassion fatigue and factors associated with symptoms to establish preventive strategies or evidence-based interventions.	Compassion fatigue was significantly higher among nursing workers (^¶¶^p=0.004) and physicians (^¶¶^p=0.022) compared to the remaining health workers.	2C^‡‡‡‡^
Cai H, et al./Medical Science Monitor: International Medical Journal of Experimental and Clinical Research/SCOPUS‡/(2020)^(^ 27 ^)^	Observational cross-sectional study/534 participants/China.	To investigate the impact of coping strategies adopted by the medical staff working in the front line in the province of Hunan, adjacent to the Hubei province, during the COVID-19^¶^ outbreak between January and March 2020.	The nursing staff experienced more significant nervousness and anxiety than the other groups (^¶¶^p=0.02). The physicians were unhappier with the overtime work performed during the COVID 19^¶^ outbreak than the other health workers (^¶¶^p=0.02). The main factors associated with stress were concerns with personal safety (^¶¶^p<0.001), concerns with the family (^¶¶^p<0.001), and concerns with the death of patients (^¶¶^p=0.001).	2C^‡‡‡‡^
Bostan S, et al./Electronic Journal of General Medicine/SCOPUS‡/(2020)^(^ 28 ^)^	Cross-sectional study /736 health workers /Turkey	To show how health workers assessed working conditions in the Turkish population and the fight against COVID-19^¶^ and whether their work in risk environmental and abnormal conditions affected their anxiety levels.	Assessment of the participants' working conditions (3.17±0.827) shows that their participation in social conditions (3.24±0.739) was moderate, though anxiety levels were high (4.36±0.841). A low and negative relationship was found between working conditions and anxiety levels (^‡‡‡^ r=-0.194) and social conditions (^‡‡‡^ r=-0.105).	2C^‡‡‡‡^
Liu CY, et al./Epidemiology & Infection/ WoS||/(2020)^(^ 29 ^)^	Descriptive cross-sectional study/512 workers/China	To verify the anxiety levels of health workers in the front line and identify the risk factors for anxiety in China during the COVID-19^¶^ pandemic.	The average score for anxiety was significantly higher in the medical team directly treating confirmed cases than among those who did not (41.11±9.79 vs. 38.83±8.38, ^¶¶^p= 0.007).	2C^‡‡‡‡^
Zhu J, et al./Frontiers in Psychiatry/SCOPUS‡/(2020)^(^ 30 ^)^	Descriptive cross-sectional study /165 workers/China	To investigate the prevalence and factors influencing anxiety and depression symptoms in the medical staff in the front line in the fight against the novel coronavirus pneumonia in Gansu.	The prevalence of anxiety and depression symptoms among the physicians was 11.4% and 45.6%, respectively, and 27.9% and 43.0% among nurses, respectively.	2C^‡‡‡‡^
Shacham M, et al./International Journal of Environmental Research and Public Health/ SCOPUS‡/ (2020)^(^ 31 ^)^	Cross-sectional study /338 dentists and dental hygienists /Israel.	To assess the association of COVID-19^¶^ and psychological factors with psychological distress in the oral care team during the COVID-19^¶^ outbreak.	A high risk of psychological distress was found in 11.5% of the sample (^§§§^n=39). High psychological stress was found among those with a background disease (^†††^OR=3.023 (||||95% CI: 1.186-7.705;^¶¶^p=0.021), fear of contracting COVID-19^¶^from a patient (^†††^OR=2.110 (||||95%CI: 1.236-3.603; ^¶¶^p=0.006) and greater subjective overload (^†††^OR=1.073 (||||95%CI: 1.010-1.141); ^¶¶^p=0.022).	2C^‡‡‡‡^
Mo Y, et al./ Journal of Nursing Management/SCOPUS‡/(2020)^(^ 32 ^)^	Cross-sectional study/180 nurses/China.	To identify the stress levels faced by Chinese nurses supporting the fight against the COVID-19^¶^ infection in Wuhan and verify which factors are relevant in developing psychological interventions directed to Chinese nurses to adjust to public health emergencies.	The total score for stress load (SAS^||||||^) was 32.19±7.56 points, which is higher than the national standards (29.78+0.46); the difference is statistically significant (t=4.27, p<0.001).	2C^‡‡‡‡^

* EMBASE = Excerpta Medica Data-base;

† MEDLINE-PubMed = Medical Literature Analysis and Retrieva

l System Online - National Center for Biotechnology Information;

‡ SCOPUS = Elsevier's database;

§ CINAHL = Cumulative Index to Nursing and Allied Health Literature;

|| WoS = Web of Science;

¶ COVID-1 9 = Coronavirus Disease 2019;

** NCP *=* Novel Coronavirus Pneumonia;

†† SARS-CoV-2 = Severe Acute Respiratory syndrome Coronavirus 2;

‡‡ PPE = Personal Protective Equipment;

§§ RR = Relative Risk;

|||| CI = Confidence Interval;

¶¶ p = p value;

*** IgG = Immunoglobulin G

††† OR = Odds Ratio;

‡‡‡ r = r value

§§§ n = absolute number;

|||||| SAS = Self-Rating Anxiety Scale;

¶¶¶ EL = Evidence Level according to the Oxford Centre for Evidence-based Medicine;

**** Level of Evidence 2B = Cohort Study;

†††† Level of Evidence 4 = Case report;

‡‡‡‡ Level of Evidence 2C = Observation of therapeutic results, ecological studies

## Discussion

The findings are presented according to two thematic categories: 1) Risk of contamination and occupational exposure among health workers providing care to patients infected with COVID-19, which was addressed by five papers, and 2) Risk of psycho-emotional illness among health workers providing care to patients infected with COVID-19, addressed by 14 papers.

### Risk of contamination and occupational exposure among health workers providing care to patients infected with COVID-19

COVID-19 is a highly contagious infection, and health workers are at a greater risk of contamination due to their exposure when providing direct care to patients with a confirmed or suspect diagnosis of COVID-19. The likelihood of health workers becoming infected is related to the duration, degree, and route of exposure to patients with COVID-19 and the amount of inhaled viruses^(^ 14 ^)^.

The authors^(^ 14 ^)^ verified that the risk of contamination was related to the profession of the workers in the hospital affiliated to the Jianghan University in Wuhan, China. The risk among nursing workers was related to the duration of exposure to infected patients. Considering that the workers have the same work routine, protection was related to the regular and correct use of PPEs and proper removal of PPEs. The risk among the physicians was related to not wearing or improperly wearing PPEs during occupational exposure, while physical protection was related to decreased exposure to the pathogen. Regardless of the profession, health workers providing pre-hospital care were at a higher risk of exposure and contamination for not being aware of the type of pathology patients presented and, consequently, did not wear proper PPE during work.

Protective clothing, shoe covers, caps, masks, and gloves associated with rigorous hygiene of hands and environment prevent and decrease infection risk. Additionally, hospitals should enhance the control and management of hospital infection, create isolated wards that meet sanitary standards, and improve the training of medical personnel regarding protective, disinfection, and isolation measures. In summary, proper protection is the most significant barrier to preventing the contamination of health providers exposed to COVID-19^(^ 14 ^)^.

In this sense, a study conducted in China^(^ 15 ^)^ highlights that the physicians and nurses directly working with patients infected with COVID-19 who did not properly wash their hands after having contact with these patients were at the greatest risk of becoming infected with COVID-19. Those facing longer working hours, especially in high-risk wards, providing care to infected patients in critical conditions, were also at a greater risk of contamination. The reason is that patients in these conditions require greater assistance, which results in workers being more exposed to aerosol-generating procedures^(^ 15 ^)^.

Effectiveness linked to the appropriate use of PPEs and adherence to rigorous sanitation measures in a tertiary hospital in Germany is reported to protect health providers against the spreading of COVID-19 from patients with a confirmed or suspected diagnosis^(^ 16 ^)^. Sensitization regarding the risk of COVID-19 infection is crucial, even in wards not admitting infected patients considering contagion intensity and uncertainty regarding signs and symptoms presented by patients.

Authors^(^ 16 ^)^ made an important consideration regarding the limitations of the diagnosis obtained by nasopharyngeal swabs. Of the five workers (100%) with SARS-CoV-2-IgG antibodies detectable in the serology, four (80%) presented a negative RT-PCR nasopharyngeal swab for COVID-19, and one (20%) was asymptomatic. Asymptomatic workers reported COVID-19-associated manifestations in the last three months, including headache (40%) and sneezing (40%); general malaise, anosmia, while fever was observed in one case only. None of the individuals reported cough, sore throat, or dyspnea.

There was difficulty in identifying the route of transmission of three workers (60%) who suspected of having being infected after being exposed to infected patients who were not protected; the route of infection was unknown in two cases (40%)^(^ 16 ^)^. Adherence to sanitary standards and social distancing is crucial, considering that asymptomatic infections or contamination from unknown sources remain routes of transmission and contamination in hospitals and social settings.

The authors^(^ 17 ^)^ of a study conducted in some Latin American countries report that 74.7% of 936 (100%) health workers, mainly physicians and nurses, accessed COVID-19 diagnosis and treatment algorithms. The frequency in which health workers accessed essential PPEs during the COVID-19 pandemic was: 91.1% wore disposable gloves, 67.3% wore disposable scrubs, 83.9% disposable masks, 56.1% wore N95 masks, and 32.6% wore face shields. The workers’ perception is that they received insufficient support from medical institutions and public health authorities to fight the COVID-19 pandemic.

These results alert to the need to urgently implement adequate protection and support strategies for health workers during the pandemic. However, as stated by the authors^(^ 17 ^)^, these findings are limited and cannot be generalized as they originate from a cross-sectional study in which an intentional sample composed of workers providing care to critical patients with varied diagnoses was used. Nonetheless, these results reinforce the importance of health facilities performing internal assessments to prevent and minimize the contamination of health workers exposed to diverse pathogens.

On the one hand, the use of PPEs is intended to protect health workers from the COVID-19 infection; on the other hand, this equipment’s prolonged use may cause discomfort and worse previous pathological conditions^(^ 18 ^)^. Additionally, 158 (100%) health workers report increased use of PPEs since the COVID-19 outbreak in Singapore^(^ 18 ^)^. On average, the interviewees wore an N95 mask for 18.3 days, 5.9 hours a day on average; 96.8% of the interviewees wore protective goggles. The authors^(^ 18 ^)^ verified that 87.5% of the participants reported a feeling of pressure or heaviness on the affected sites, characterized by some as a throbbing (n=15, 11.7%) or pulling pain (0.8%). The study’s participants reporting pre-existing primary headaches and those working in the emergency room were more likely to develop headaches associated with the prolonged use of PPEs.

Based on the previous discussion^(^ 14 ^,^ 18 ^)^, it is essential to pay attention to the risk of health workers acquiring illnesses other than the COVID-19 infection and protect these workers’ integral physiological and psycho-emotional health. Hence, the appropriate use of PPEs should be assessed and whenever possible, rotate health workers providing care to patients with a confirmed or suspected COVID-19 diagnosis to decrease the time workers are exposed to the disease and minimize the prolonged use of PPEs.

### Risk of psycho-emotional illness among health workers providing care to patients infected with COVID-19

The mental health of medical and nursing teams has been considerably challenged during the COVID-19 pandemic. Health workers gradually presented psychological distress during the pandemic; fear and anxiety preceded depression, psychophysiological changes, and post-traumatic stress. Being isolated, working in sites with a high risk of contamination, and having contact with infected patients are common causes of trauma, negatively impacting workers’ mental health and triggering psycho-emotional illnesses^(^ 21 ^)^.

Prolonged exposure to adverse experiences arising from professional practice and care delivery may trigger fear and trauma, indirectly absorbed by workers during inter-relations established at work. These feelings are typical of vicarious trauma or secondary traumatic stress. Described as a sudden biopsychosocial adverse response that causes severe physical and mental problems, vicarious trauma is experienced by those in close contact with patients and absorb their suffering, as is the case of health workers providing care to individuals with a confirmed or suspected COVID-19 diagnosis^(^ 19 ^)^.

The results of the first study^(^ 19 ^)^ addressing nurses’ psychological status, regardless of whether they worked in the care and control of COVID-19 infections in China, suggest that the workers were experiencing vicarious trauma due to the pandemic. Note that indirect vicarious trauma among workers not working in the front line was even more severe than among those directly providing care to patients with COVID-19 (64 *vs*. 75.5, p<0.001).

Another study^(^ 21 ^)^ developed in China to assess mental disorders among 994 physicians and nurses reports that 34.4% presented mild mental disorders, 22.4% moderate disorders, and 6.2% presented severe disorders. Workers with a high level of psychological distress had been more frequently exposed to patients infected with COVID-19. Exposure risk factors (having a confirmed or suspected diagnosis among patients, themselves, family members, friends, co-workers, neighbors, and co-residents) affected these individuals’ mental health and their physical health self-perception. Health workers with higher levels of mental problems manifested a more urgent desire to seek a psychotherapist or psychiatrist’s assistance.

Authors^(^ 29 ^)^ assessing the anxiety levels of 512 health workers working in the front line of the COVID-19 pandemic in China verified that 10.35% experienced mild anxiety, 1.36% moderate anxiety, and 0.78% experienced severe anxiety. The average score of anxiety was higher among workers who directly cared for confirmed cases than those who did not (41.11±9.79 vs. 38.83±8.38, p=0.007). Providing direct care to patients infected with COVID-19 appeared an independent risk factor for increased anxiety scores (β=2.280, CI 0.636–3.924; p=0.0068). Health workers in quarantine in Hubei and also cases with a suspected diagnosis presented increased anxiety scores.

Regarding anxiety and depression symptoms among health workers providing care to patients infected with COVID-19, nurses presented higher levels of anxiety (26.88% *vs*. 14.29% p=0.039^(^ 22 ^)^; 4.36±0.841^(^ 28 ^)^) and depression symptoms (54[7.1%] *vs*. 24[4.9%]; P=0,01^(^ 20 ^)^) than physicians^(^ 30 ^)^. Other factors, however, such as being a woman (OR: 1.94; 95%CI, 1.26-2.98; P=0.003^(^ 20 ^)^), the level of hospital complexity (depression: OR: 1.65; 95%CI, 1.17-2.34; P=0.004, and anxiety: OR: 1.43; 95%CI, 1.08-1.90; P=0.01^(^ 20 ^)^), and COVID-19 service lines, affected the behavior of events that varied in accordance to the pandemic epicenters, as in the case of the city of Wuhan, China^(^ 20 ^)^.

The analysis shows an interaction between anxiety levels presented by the health staff during the different demands of patients with COVID-19, resulting in increased stress levels and decreased self-efficacy and sleep quality. Additionally, social support as a protective mechanism during the pandemic appears relevant^(^ 23 ^)^.

The feelings and symptoms presented by health workers during the COVID-19 outbreak revealed that the main stressors were linked to personal safety (p<0.001), concerns with family (P<0.001), and concerns with the death of patients (p=0.001). The variables age (>50 years old) and being a woman presented significant differences when compared to other working groups, while coping strategies (p=0.04) depending on sex^(^ 27 ^)^ were the most effective in decreasing stress.

Regarding the rates of burnout syndrome among workers from the multi-professional team at the cancer hospital of Hubei, China, a significantly lower frequency of the burnout syndrome was found^(^ 25 ^)^ among front line workers compared to workers in other hospital services (13% and 39%; p<0.0001, respectively). Despite a similarity in terms of already known risk variables (marital status and experience in years), the factors that possibly explain this behavior would be having a sense of control over situations and at work, which helped prevent burnout in the health staff. Another study^(^ 26 ^)^ reports that compassion fatigue was significantly greater among nursing workers (p=0.004) and physicians (p=0.022) compared to the remaining health workers, highlighting that women (p=0.014) and single individuals (p=0.039) were at a higher risk of developing compassion fatigue.

An Israeli study addressing the association of COVID-19 and psychological factors with psychological distress in the oral care staff during the pandemic reports an 11.5% prevalence risk of psychological distress in addition to an association between psychological stress and comorbidities, fear of contagion, and subjective overload (OR=3.023, p=0.021; OR=2.110, p=0.006 and OR=1.073, p=0.022, respectively). On the other hand, a lower risk of psychological distress among dentists and oral hygienists was associated with commitment and self-efficacy (OR=3.023, p=0.021 and OR=0.889, p=0.005, respectively). The latter refers to the development of support resources to cope with situations and the consequences of high psychological distress, as is the case of a pandemic^(^ 31 ^)^.

The Chinese studies also assess stress levels among health workers in contact with patients with a confirmed or suspected diagnosis of COVID-19. The authors reported significantly high levels of stress [Perceived Stress Scale-14 (PSS-14): 28], higher than safe criteria (PSS: 25/26)^(^ 24 ^)^, and a correlation between stress and anxiety levels when fighting COVID-19 infection among Chinese people^(^ 32 ^)^. Additionally, other factors negatively affect stress levels among health workers, such as their position and experience in years, anxiety and depression levels, protective measures, and hospital contact history^(^ 24 ^)^.

Psychological counseling to prevent, alleviate, or treat increased psycho-emotional illnesses among health workers is essential during a pandemic, regardless of the sector one works or whether s/he provides assistance to patients with a confirmed or suspected infection or not.

Regarding limitations, most were cross-sectional studies with issues inherent to the design, sample sizes, and sampling, which restrict the generalization of results to similar populations or impede drawing cause and effect conclusions.

## Conclusion

This integrative review enabled mapping the scientific literature addressing the potential effects of providing care to patients infected with COVID-19 on workers’ health. According to the Oxford classification, most papers presented an evidence level equal to 2C for observational studies. Thus, future studies with a higher level of evidence are needed to provide more robust evidence or validate current findings, exploring the interaction between a potential causal nexus of the occupational risk of becoming infected with COVID-19 during the pandemic.

The results present the primary occupational risks described during the pandemic of the novel coronavirus. These are biological and psychosocial risks that are intricate in a care delivery historical past arising from direct care delivery, which obviously became more acute within the context of providing care during the pandemic.

The studies first showed the occupational risk of contamination and biological exposure of health workers, emphasizing the relevant protection of correctly and effectively wearing PPEs, in addition to the impact of proper hand hygiene and the hospital environment sanitation. Secondly, the studies report a relationship between the pandemic and increased levels of stress, anxiety, depression, and compassion fatigue.

In this sense, studies developed during the current pandemic show an urgent need to assess the risks to which health workers are exposed during occupational activities. Illnesses go beyond the physical and physiological spheres, negatively impacting the individuals’ psycho-emotional conditions, compromising their wellbeing, quality of life, and work performance.

Similarly, processes within the work environment and care delivery context to deal with this public health problem include the hospital setting’s management, highlighting the importance of health managers to implement strategies to manage occupational risks in health services.

Therefore, investment is needed to prepare, assist, and provide mental health devices to protect and care for future multidisciplinary teams who may be surprised with the need to be on the front lines to combat the outbreak of infectious diseases.

At the same time, it is vital to ensure workers are qualified, that there are sufficient and quality PPEs to protect the health and wellbeing of workers, sufficient personnel to enable multidisciplinary teams to take turns between work periods and breaks, to minimize impacts on the health of workers arising from excessive working hours.

These results suggest a need to implement structured, evidence-based interventions addressing psychosocial risks, supported by guidelines and policies recommended by the Ministry of Health and international health agencies as an indispensable tool to preserve the health of workers while also supporting the formulation of mental health policies necessary in critical times such as those experienced within the COVID-19 pandemic.

Information and follow-up systems are needed to address the widely discussed occupational risk factors and their interaction with therapeutic strategies to produce more comprehensive data that will effectively protect the health of multi-professional teams working in the front line of the COVID-19 outbreak.
